# Development of revised ResNet-50 for diabetic retinopathy detection

**DOI:** 10.1186/s12859-023-05293-1

**Published:** 2023-04-19

**Authors:** Chun-Ling Lin, Kun-Chi Wu

**Affiliations:** Department of Electrical Engineering, Ming Chi University of Technology, No. 84, Gongzhuan Rd., Taishan Dist., New Taipei City, 243 Taiwan

**Keywords:** Diabetic retinopathy (DR), Deep learning, Xception, Alexey, VggNet-, VggNet-16, ResNet-50

## Abstract

**Background:**

Diabetic retinopathy (DR) produces bleeding, exudation, and new blood vessel formation conditions. DR can damage the retinal blood vessels and cause vision loss or even blindness. If DR is detected early, ophthalmologists can use lasers to create tiny burns around the retinal tears to inhibit bleeding and prevent the formation of new blood vessels, in order to prevent deterioration of the disease. The rapid improvement of deep learning has made image recognition an effective technology; it can avoid misjudgments caused by different doctors’ evaluations and help doctors to predict the condition quickly. The aim of this paper is to adopt visualization and preprocessing in the ResNet-50 model to improve module calibration, to enable the model to predict DR accurately.

**Results:**

This study compared the performance of the proposed method with other common CNNs models (Xception, AlexNet, VggNet-s, VggNet-16 and ResNet-50). In examining said models, the results alluded to an over-fitting phenomenon, and the outcome of the work demonstrates that the performance of the revised ResNet-50 (Train accuracy: 0.8395 and Test accuracy: 0.7432) is better than other common CNNs (that is, the revised structure of ResNet-50 could avoid the overfitting problem, decease the loss value, and reduce the fluctuation problem).

**Conclusions:**

This study proposed two approaches to designing the DR grading system: a standard operation procedure (SOP) for preprocessing the fundus image, and a revised structure of ResNet-50, including an adaptive learning rating to adjust the weight of layers, regularization and change the structure of ResNet-50, which was selected for its suitable features. It is worth noting that the purpose of this study was not to design the most accurate DR screening network, but to demonstrate the effect of the SOP of DR and the visualization of the revised ResNet-50 model. The results provided an insight to revise the structure of CNNs using the visualization tool.

## Background

Diabetes is one of the most serious and common chronic diseases in the world; it causes life-threatening, disabling, and costly complications, and reduces life expectancy. The 9th edition of the IDF report shows that there was a prevalence of 9% (463 million adults) in 2019, and the 10th edition of IDF estimates that there were 537 million people living with diabetes worldwide in 2021 [1], with its global prevalence estimated to be over 10%. Furthermore, the number of patients suffering from diabetes mellitus is expected to increase significantly. Diabetic retinopathy (DR) is a potentially blinding complication of diabetes mellitus [2]. DR causes impaired vision and may even lead to blindness if it is not diagnosed in early stages. Thus, retinal examinations remain a primary component of DR management, and they are essential for reducing the long-term consequences of the disease. It is difficult but of the utmost importance to recognize and treat DR, to avoid the more serious risk of permanent blindness in at-risk individuals [3].

Deep learning (DL) is a multilayer neural network learning algorithm that has emerged in recent years. DL has brought a new perspective to machine learning, leading to significant advances in artificial intelligence and human–computer interactions [4]. The rapid improvement of deep learning has made image recognition an effective technology; it can avoid misjudgments caused by different doctors’ standards, and help doctors to diagnose the condition more quickly. Thus, many recent studies have applied deep learning and image recognition technology to the recognition of DR [5–11].

Khan et al. [5] proposed a VGG16 model, spatial pyramid pooling layer (SPP), and network-in-network (NiN) to speed up the convergence of the training model due to the lowest parameters (45,486,280 parameters). This proposed VGG-NiN model was trained and tested using a Kaggle dataset in a GPU environment. The VGG-NiN model revised the structure of the VGG16 to improve the efficiency and accuracy of the detection of DR (recall of 55.6%, precision of 67%, specificity of 91%, and F1-score 59.6%). Pratt et al. [6] developed a CNN model with a data augmentation method that extracted the special features, such as micro-aneurysms, exudate, and hemorrhages, to predict DR. In generation, data augmentation was the most common method utilized to enlarge the datasets and increase the robustness of the trained network to variations in the input image. In other words, DL can be trained for variations of the original images to compensate for variations in the images taken due to inconsistent environments, equipment, and photographers [12]. Furthermore, the number of healthy fundus images in the Kaggle dataset was much more than the ones with DR. Therefore, the DR dataset was usually augmented to almost the same size as the No DR dataset. They trained and tested the CNN model in a GPU environment and adopted the Kaggle dataset to test the performance of the proposed method.

The proposed CNN could obtain a sensitivity of 95% and an accuracy of 75% in 5000 validations. Qummar et al. [7] adopted a Kaggle dataset to train the ensemble of five common DL models (Resnet50, Inceptionv3, Xception, Dense121, Dense169 and Xception) in a GPU environment and obtained the accuracy of 80.8% for the classification of DR. The results of the ensembled model showed that the proposed model required computation power and was better than the previous state-of-the-art methods (CNN model [6]). Jabbar et al. [8] proposed the data augmentation operations on DR to solve the data misbalancing problem in the Kaggle dataset (35,126 images). Then, they extracted the features from the fundus images using the pre-trained network VGG16 and adopted the transfer learning method to enhance the performance in classifying DR.

The proposed model was trained and tested using the Kaggle dataset in a CPU (Intel(R) 3rd Generation Core (TM) i5-3470) environment. The results of the proposed method obtained an accuracy of 96.6%. Asia et al. [9] adopted the OpenCV and Keras libraries for the preprocessing, regularization, and augmentation steps of data augmentation. Then, they adopted three common neural network models, including ResNet-101, ResNet-50, and VggNet-16, to detect DR within the Hospital Ophthalmology (XHO) datasets. The previous study compared these three networks to determine the best one for DR detection. The results showed that ResNet-101 achieved an accuracy of 98.88% and that ResNet-101 had better accuracy than ResNet-50 and VggNet-16 in terms of DR classification. In order to avoid the time and resource consumed problem in DL, Mohammadian et al. [10] proposed transfer learning, such as feature extraction and fine-tuning to the Inception-V3 and Xception models, in order to classify the Kaggle dataset into two classes. They also adopted the data augmentation to reduce overfitting of the DL.

The Kaggle dataset was augmented by shifting, rotating, and flipping the images in the middle of each training. They adopted software packages (Tensorflow, Numpy, h5py, Scikit-learn, OpenCv, and Keras) to implement the proposed models in the Intel i7 core CPU environment, which was considerably advantageous compared to above CNN training hardware requirements. The results showed that fine tuning the last two blocks of Inception-V3 model utilizing RELU as the activation function can obtain about 87% accuracy on the test dataset. Wan et al. [11] adopted AlexNet, VggNet, GoogleNet, ResNet, analyzing how well these models do with the DR image classification. Furthermore, they suggested that an image of low quality would produce inaccurate results, and the preprocessing is an important operation in regards to improving image quality. In addition, normalization schemes and data augmentation can be adopted to preprocess because of the noisy data and the limited number of data sets. Also, the fundus images can be cropped to a smaller size in order to eliminate the extra areas. Thus, they adopted the nonlocal means denoising (NLMD) baseline normalization scheme, rotated, randomly stretched and flipped methods to increase fundus images. The results showed that the overall accuracy of classification was poor when CNN architectures were paired with randomly initialized parameters (AlexNet: 73.04%, VggNet-s:73.66%, VggNet-16:48.13%, VggNet-19:82.17%, GoogleNet:86.35%, ResNet:78.68%). At the same time, in the process of training, they found that there was an over-fitting phenomenon in the trained CNN models. Thus, they adopted the transfer learning and hyperparameter-tuning methods to improve the performance of classifying the fundus images and working out the over-fitting problem.

The results of the APTOS 2019 Blindness Detection study show that the recent methods can obtain a DR grading with an accuracy of between 75 and 85% [13]. The current deep learning framework for detecting and grading DR is ResNet-50 [14, 15]. However, the disadvantages of ResNet-50 are overfitting and fluctuations in accuracy, which then affect the accuracy of detecting DR. In summary, the common drawbacks from DL models include 1. limited datasets, 2. twisted and blurred images, 3. overfitting models, and 4. limited computing power.

The aim of this study is to apply preprocessing methods and a revised structure of ResNet-50, to improve its performance in detecting DR. This study proposes using the standard operation procedure (SOP) to process the fundus image. In each generation, ResNet-50 adopts the adaptive learning rating to adjust the weight of layers. In order to change the structure of ResNet-50, this study adopts the visualization tool to obtain the suitable features, instead of original features form ResNet-50. The results show that the performance of the revised ResNet-50 is better than the original ResNet-50. Finally, this study implements the proposed system using JavaScript, so that users can upload the fundus image to the website and obtain the DR results.

## Results

The DR dataset used in this study, obtained from Kaggle, includes 35,126 fundus images, of which 25,805 are normal (without disease), and only 9,321 exhibit DR. The training data include 1,500 normal fundus images, labeled as 0, and 1,500 DR images, labeled as 1. In addition, the validation and testing data use 300 fundus images individually, which are different from the training data. Accuracy and loss function are examined to evaluate the performance of the deep learning system:

1 $$accuracy = \frac{TP + TN}{{TP + TN + FP + FN}}$$

2 $$loss \left( {cross entropy} \right) = \frac{ - 1}{N}\mathop \sum \limits_{1}^{N} y_{i} *\log \left( {\widehat{{y_{i} }}} \right) + (1 - y_{i} )*\log \left( {1 - \widehat{{y_{i} }}} \right)$$

where TP is true positives, TN is true negatives, FP is false positives, and FN is false negatives. N is the number of samples, ${y}_{i}$ is the actual output, and $\widehat{{y}_{i}}$ is the predicted output.

### L1 regularization and L2 regularization in kernel_regularizer

**Fig. 1:**
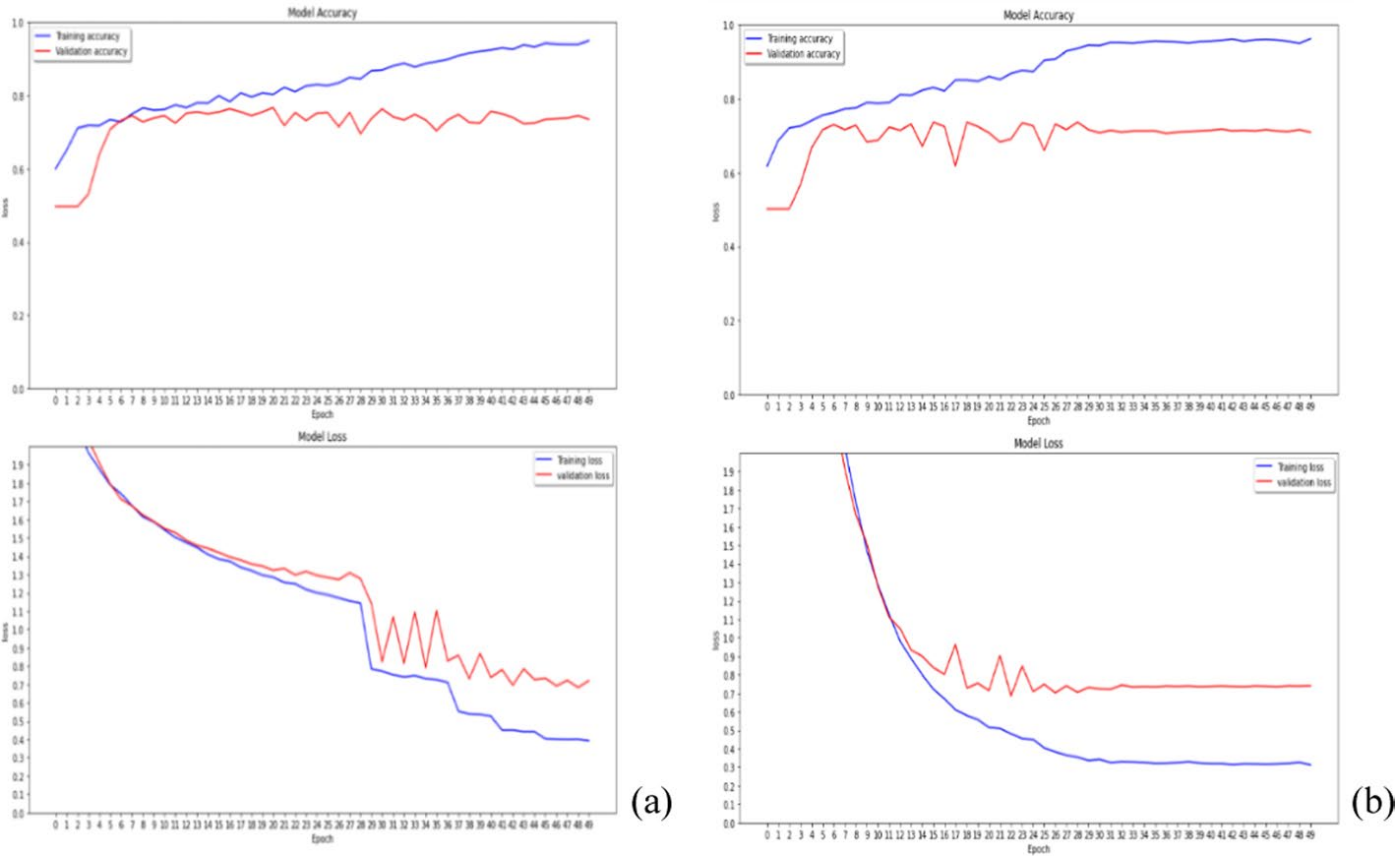
The performance of L1 (**a**) and L2 (**b**) regularization in kernel_regularizer

The L1 regularization and L2 regularization apply a penalty on the layer’s kernel individually, to evaluate which regularization is suitable for the layer’s kernel, as shown in Fig. 1. Although the performance of two regularizations is similar, the training model’s performance using L1 continues to increase slowly in the later epochs, which may cause overfitting problems. Thus, L2 regularization is adopted to apply a penalty on the layer’s kernel.

### L1 regularization and L2 regularization in activity_regularizer

**Fig. 2:**
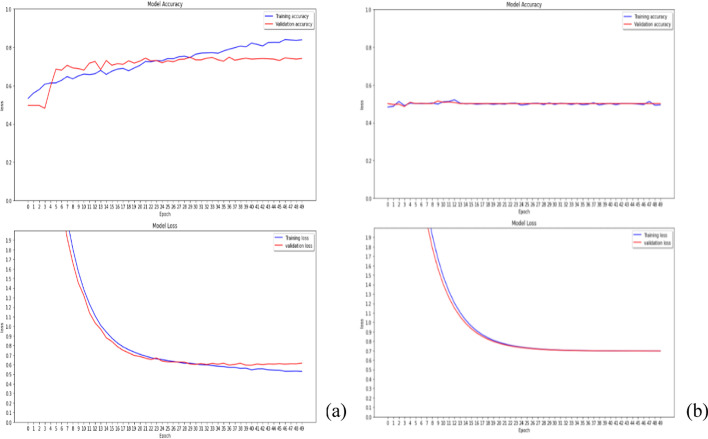
The performance of L1 (**a**) and L2 (**b**) regularization in activity_regularizer

The L2 regularization is adopted to apply a penalty on the layer’s kernel. Next, L1 regularization and L2 regularization apply a penalty on the layer’s output, as shown in Fig. 2. The result shows that L1 regularization in activity_regularizer can perform better than L2, because the former’s results give higher accuracy and lower loss in validation data.

### Adaptive learning rate in ResNet-50

**Fig. 3:**
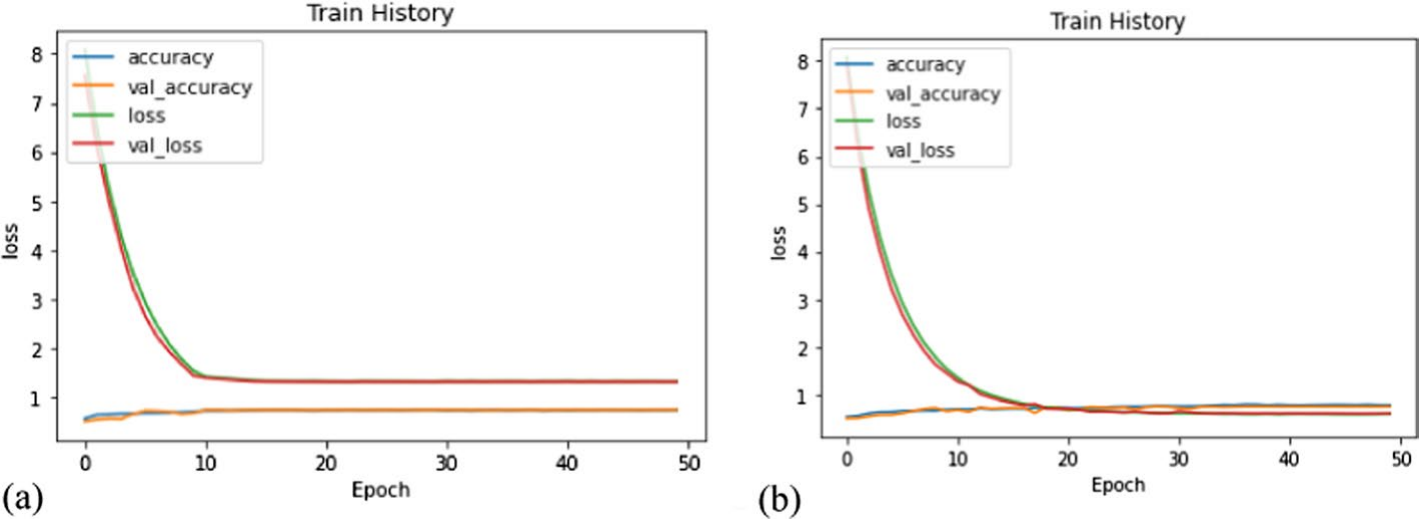
The adaptive learning rate with factor = 0.1 (**a**) and factor = 0.5 (**b**)

In general, the learning rate in deep learning is fixed. This study adopts the adaptive learning rate, instead of fixed learning rate. The common value of factors in Eq. (3) is 0.1, which causes a steep decrease in the learning rate, with the loss value remaining at 1 (Fig. 3a). That is, the coverage of model focuses on early epochs. This study selects the initial learning rate ($lr$) = 0.01 and factor = 0.5. Figure 3b shows that the minimum learning rate can reach 10^–11^, and the performance can increase when the training model adopts the proposed designed parameters.

### Suitable features from conv5_block1_out and conv5_block2_out in ResNet-50

**Fig. 4:**
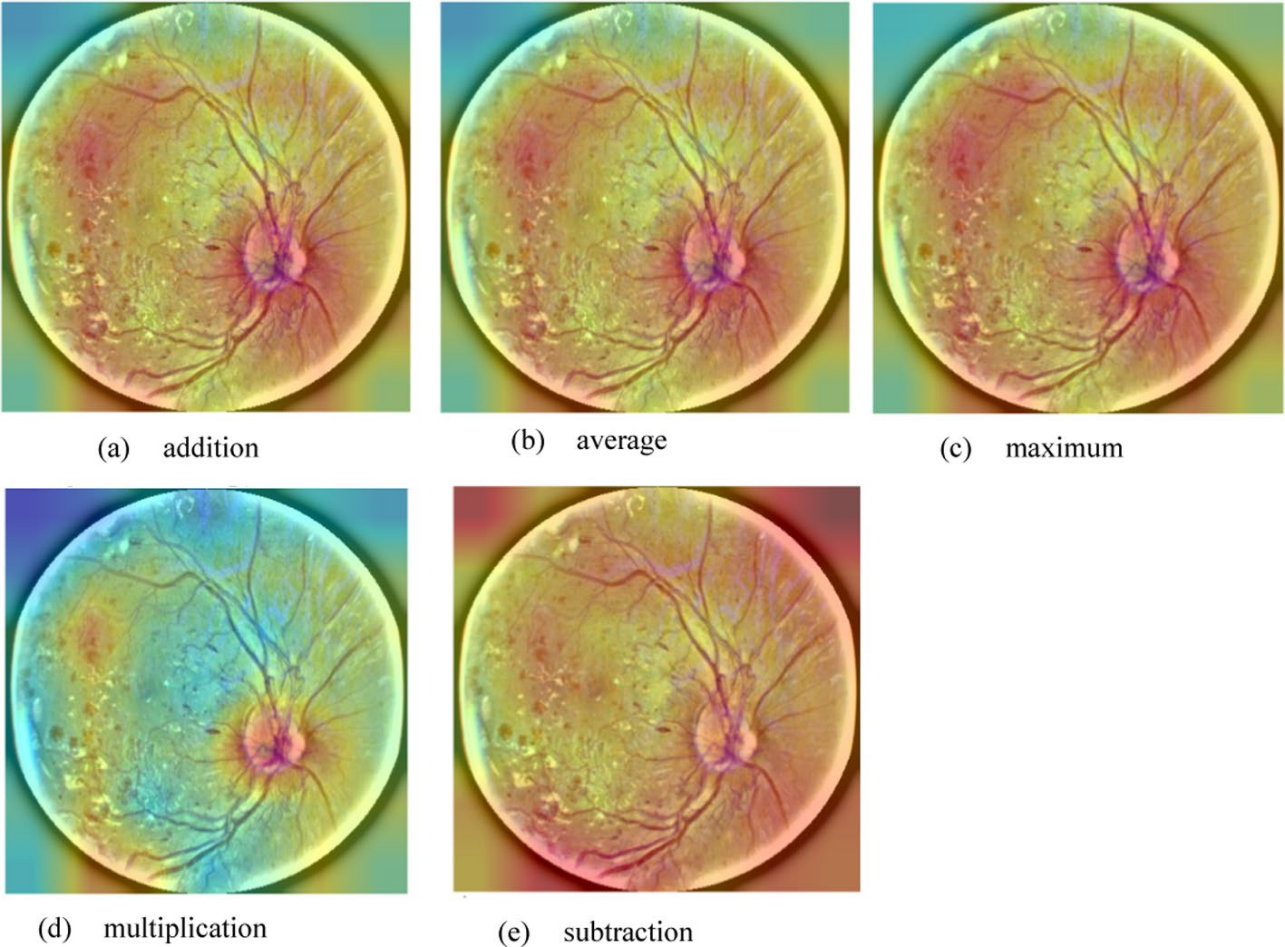
Merge the features from conv5_block1_out and conv5_block2_out using different methods: **a** addition, **b** average, **c** maximum, **d** multiplication, and **e** subtraction

The visualization tool illustrates the features from the final layer of ResNet-50, as shown in Fig. 12c, where the bleeding part in red color does not appear clearly; instead, the bleeding part can be observed in the conv5_block1_out and conv5_block2_out in ResNet-50 (Fig. 12a, b). For conv5_block1_out, peripheral color distribution is relatively average. For conv5_block2_out, the characteristic bleeding part is clearly shown. Next, this study adopts different mathematical methods (addition, subtraction, multiplication, average, and maximum) to merge the features from conv5_block1_out and conv5_block2_out, as shown in Fig. 4. The features using the subtraction method are not clear; those using addition, average and maximum are similar to each other. Features using multiplication are clearer than in other methods, and in the original layer (Fig. 12c).

This study evaluates and compares the performance of DR grading using different merging methods. In order to observe the performance of these different methods, each is performed five times independently, as shown in Table 1. The result also shows that merged features using multiplication can obtain higher accuracy than other methods.

**Table 1:** The averaged accuracy of DR grading among different methods

	Multiply	Add	Average	Maximum	Subtract
Training accuracy	0.7876	0.7892	0.8377	0.82342	**0.88048**
Training loss	0.5747	0.57436	0.5533	0.56892	0.51524
Validation accuracy	0.74326	**0.74696**	0.73142	0.74088	0.7311
Validation loss	**0.6077**	0.5979	0.615	0.61406	0.6376
Test accuracy	0.77224	0.76076	0.76076	0.7628	0.75064

The best performance is highlighted in bold

### Compare the performance of DR grading between ResNet-50 and revised ResNet-50

**Fig. 5:**
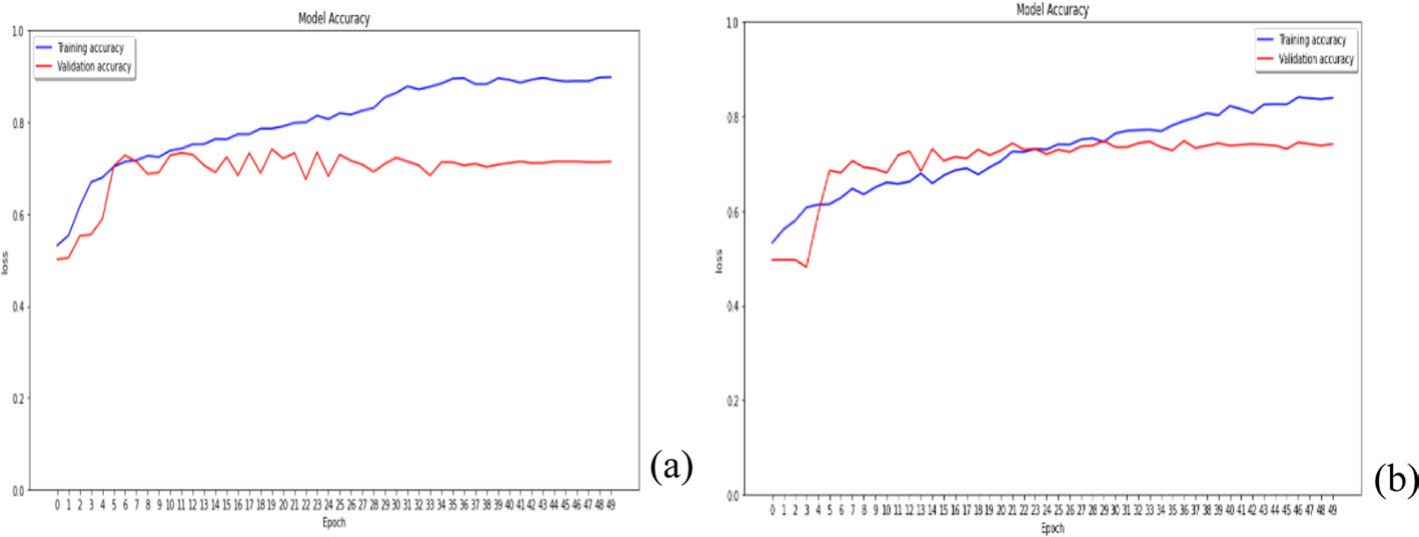
The performance of DR grading using ResNet-50 (**a**) and revised ResNet-50 (**b**)

This study compares the performance of DR grading between ResNet-50 and revised ResNet-50 (Fig. 5 and Table 2). The result shows that the revised ResNet-50 avoids the overfitting problem, deceases the loss value, and reduces the fluctuation problem. Different CNNs have overseen great achievements in regards to their good performance in image classification. This study compared the performance of the proposed method with other common CNNs models (Xception [10], AlexNet, VggNet-s, VggNet-16 and ResNet-50[11]) in the same condition as the CNN model with randomly initialized parameters featured in Table 3. In comparing the CNN models, the results indicated that there was an over-fitting phenomenon and that the overall accuracy of classification was poor. Although Mohammadian et al. [10] and Wan et al. [11] suggested that transfer learning and hyperparameter-tuning methods can improve the performance in classifying fundus images and working out the over-fitting problem, the definition of frozen and tuning in layers and blocks or activation was a special issue and should adopted the different experiments. It is worth noting that the purpose of this study was not to design the most accurate DR screening network, but to demonstrate the effect of SOP of DR and visualization of the revised ResNet-50 model. The results provided insight to revise the structure of CNNs using a visualization tool.

**Table 2:** The performance between ResNet-50 and revised ResNet-50

Model/performance	ResNet-50	Revised ResNet-50
Train accuracy	0.8981	0.8395
Train loss	0.4805	0.5287
Validation accuracy	0.7145	0.7416
Validation loss	0.6603	0.6155
Test accuracy	0.7567	0.7432
Test loss	0.6474	0.6018

**Table 3:** Classification results with randomly initialized parameters of the CNN model

Model/performance	Xception	AlexNet	VggNet-s	VggNet-16	ResNet-50	Revised ResNet-50
Test accuracy	78.60%	73.04%	73.66%	48.13%	75%	74%

Finally, the proposed DR grading system, which was trained using the Kaggle DR dataset, is applied to test its performance with the publicly available JSIEC dataset, using 144 fundus images. The averaged accuracy of DR grading is 0.9485 (loss = 0.4295), from five independent runs.

### Online DR grading system

**Fig. 6:**
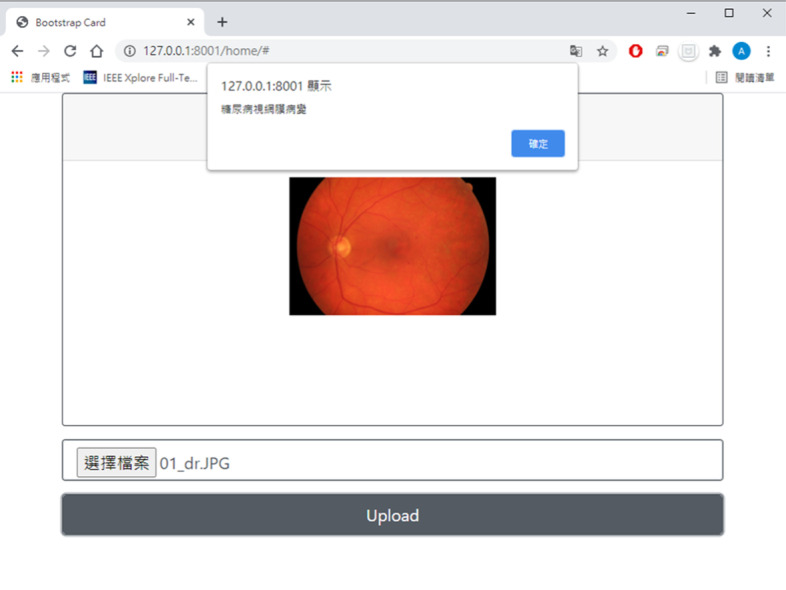
Online DR grading system

The study adopts JavaScript to “post” the fundus image to the server and obtain the result. The time between posting and obtaining the result is 1 s. Users also can drag the image to the designated area and upload it. When the user selects the upload button, they will be notified of the result in a message window, as shown in Fig. 6. The online DR grading system was only set up in the local area network (LAN) of Ming Chi University of Technology (MCUT) to test the performance of the online DR grading system to verify the system can detect the DR condition in real-time. In further work, we propose real-time online testing of the DR grading system with a public URL where users to upload a fundus image to the public URL and obtain the DR results.

## Discussion

The aim of this study was to adopt preprocessing methods and revise the structure of ResNet-50 to improve its performance in detecting DR. The various normalization and pre-processing aspects are very important for said detection. If pre-processing and normalization are not set up suitably, the training model can cause the phenomenon of overfitting easily, so, it is also very important that there is accuracy in the pre-processing method. The proposed SOP can be followed to improve the quality of fundus images (Fig. 11). In addition, this study adopted the heat map to find the characteristic convolutional layer and matched the convolutional layer with the fusion layer to define suitable features. Furthermore, adaptive learning rate and regularize methods were also adopted to improve the accuracy and reduce loss through different experiments. Figure 5 and suggested that transfer learning and hyperparameter-tuning methods can improve the performance in classifying fundus images and working out the over-fitting problem, the definition of frozen and tuning in layers and blocks or activation was a special issue and should adopted the different experiments. It is worth noting that the purpose of this study was not to design the most accurate DR screening network, but to demonstrate the effect of SOP of DR and visualization of the revised ResNet-50 model. The results provided insight to revise the structure of CNNs using a visualization tool.

Tables 2 and 3 demonstrated that the revised structure of ResNet-50 could avoid the overfitting problem, decease the loss value, and reduce the fluctuation problem. The difference between train accuracy and test accuracy (9.63%) in the revised ResNet-50 was smaller than in the original ResNet-50 (14.14%).

There exist three major limitations in this study which could be addressed in future research The DR dataset from Kaggle includes 35,126 fundus images, of which 25,805 are normal (without disease). If the number of images in the trained model was fewer, thus affecting the reliability of the image recognition model [16, 17], we could increase the number of the DR dataset for development of the Revised ResNet-50 to increase the reliability of the Revised ResNet-50. Second, if the features were only chosen from the whole fundus images, the detail and important features might miss. Thus, we can adopt U-Net [31] in the SOP and then extract the detail features of DR, such as blood clots, to improve the accuracy in detecting DR. Third, we can propose real-time online testing of a DR grading system in a public URL for users to upload a fundus image to the public URL and obtain the DR results. Thus, in future, we will further analyze more different CNN models with transfer learning and hyperparameter-tuning methods to improve the performance of classifying the fundus images.

## Conclusion

In order to predict diabetic retinopathy, this study first applied SOP to process fundus images in order to improve their quality. Then, this study proposed the revised ResNet-50 model for detecting DR: adaptive learning rating to adjust the weight of layers, regularization and change the structure of ResNet-50, which selected the suitable features from conv5_block1_out and conv5_block2_out in ResNet-50. The results of this study demonstrate that the performance of the revised ResNet-50 (Train accuracy: 0.8395 and Test accuracy: 0.7432) is better than that of the original ResNet-50 (Train accuracy: 0.8981 and Test accuracy: 0.7567) and other common CNN models (Xception, AlexNet, VggNet-s, VggNet-16). That is, the revised structure of ResNet-50 could avoid the overfitting problem, decease the loss value, and reduce the fluctuation problem. Finally, this study develops an online DR grading system using JavaScript, enabling users to upload a fundus image to the website and obtain the DR results.

## Methods

**Fig. 7:**
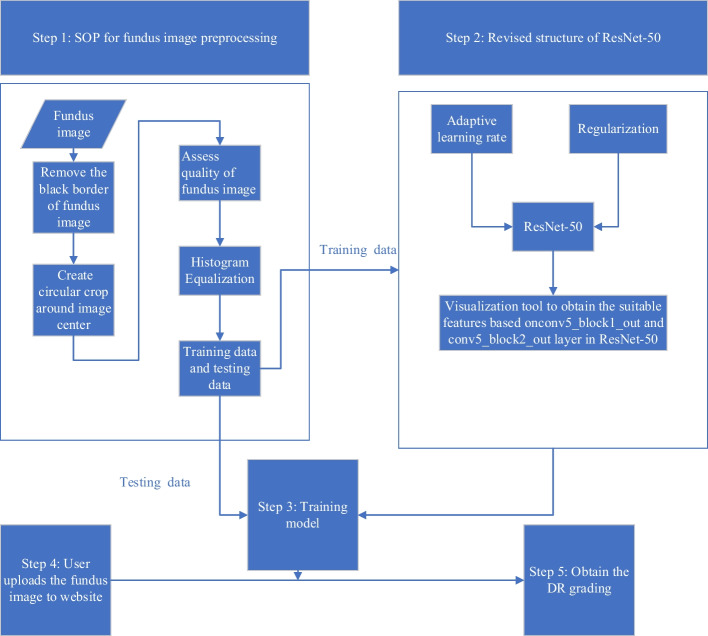
The flowchart of this study

This study proposes two approaches to designing the DR grading system: a standard operation procedure (SOP) for preprocessing the fundus image, and a revised structure of ResNet-50, which is described in the following subsection. Finally, this DR grading system is implemented in a website, to allow users to check a fundus image by themselves. Figure 7 shows the flowchart of the proposed DR grading system.

### Dataset

In order to verify the accuracy that a deep learning system will achieve with a DR dataset, the concept of training, validation, and testing must be applied. The DR dataset from Kaggle [18] includes 35,126 fundus images, of which 25,805 are normal (without disease). Only 9,321 fundus images exhibit DR, which is divided into four stages [19]: mild nonproliferative diabetic retinopathy (NPDR), moderate NPDP, severe NPDP, and proliferative diabetic retinopathy (PDR). The imbalanced proportion of normal and DR images in Big Data has been identified as one of the main challenges for the algorithms. This can commonly cause overfitting problems [20], as there is a high performance of DR grading in training data, but low performance in the testing data.

**Fig. 8:**
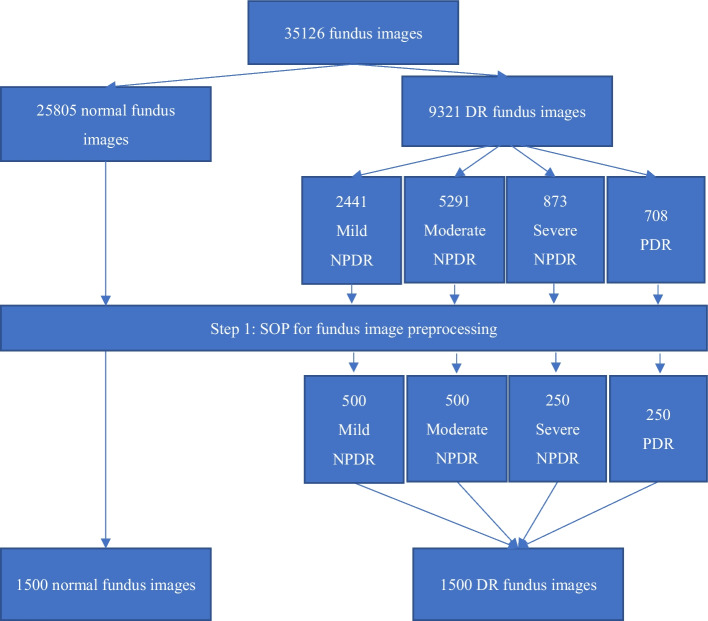
The method for selecting the training data from 35,126 fundus images

Figure 8 shows the method used to select the training data from the 35,126 fundus images. In addition, the validation and testing data also follow a similar method to select 300 fundus images individually, which are different from any images in the training data.

### SOP for fundus image preprocessing

Preprocessing methods are a very important stage in image recognition; they can be used to eliminate noise/variation in the retinal fundus image, and improve the quality and contrast of the image. Consequently, the trained modules can obtain more credible and accurate results. Therefore, the proposed SOPs (Fig. 1 step 1) for preprocessing fundus images in this study are introduced respectively.

#### Remove the black border of the fundus image

**Fig. 9:**
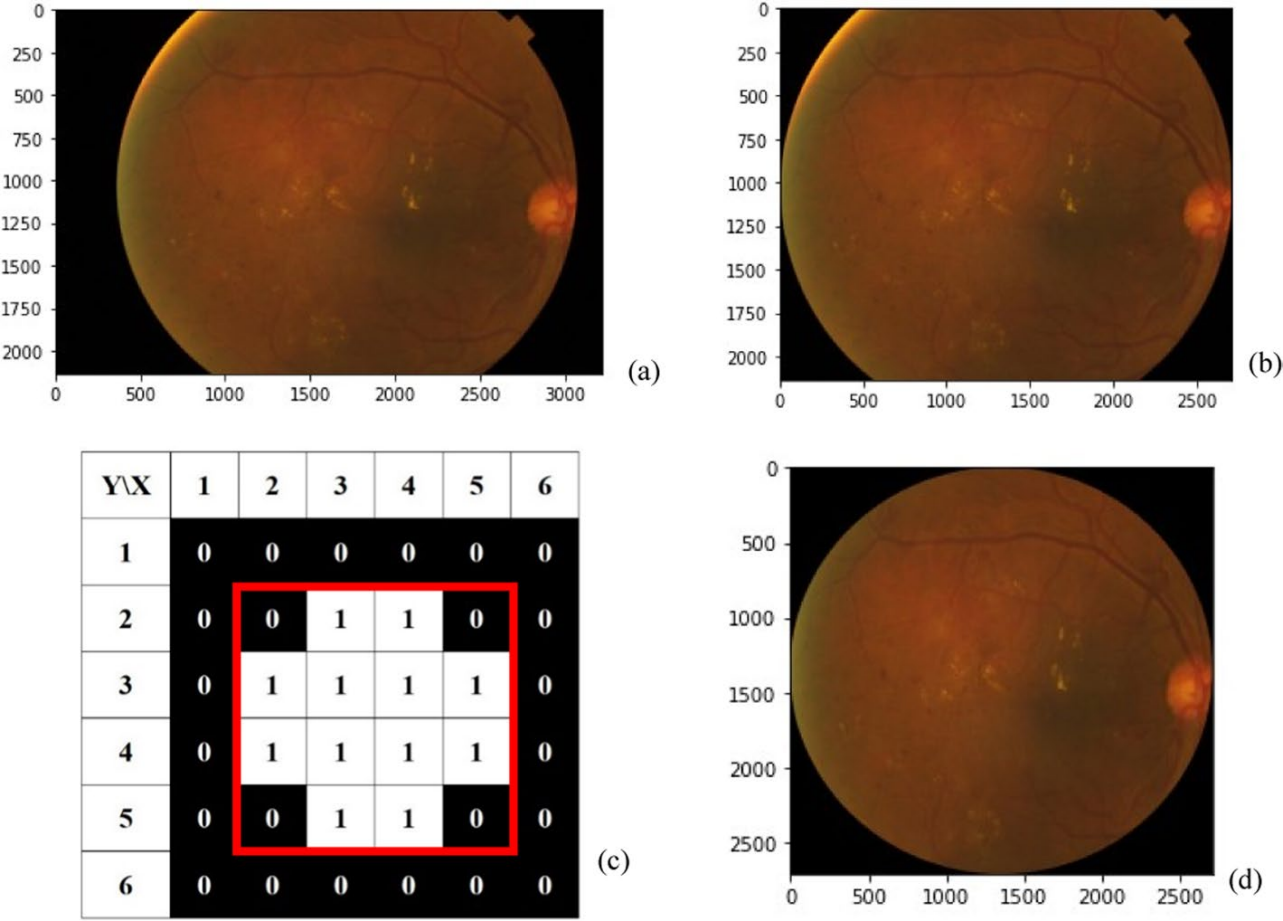
**a** The fundus image with a black border,; **b** the fundus image after removing the black areas; **c** the values in the clipping mask are used to remove black areas of the fundus image; **d** a circular crop around the center of the fundus image

Many types of fundus image are obtained from Kaggle, due to different types of fundus photography equipment and environments. For instance, the black border of the fundus image (Fig. 9a) would affect the performance of DR grading. This study adopts the auto-cropping method [21] to crop out the uninformative black areas, as shown in Fig. 9b. The auto-cropping methods, using crop_image_from_gray functions [21], are performed as follows:

1. Convert this image (RGB format) to Gray format using OpenCV library. The value of a pixel is 255 when the color of the image is white; if the color is black, the value of the pixel is 0.
2. Produce the clipping mask which contains 0 and 1 values. When the value of a pixel > tolerance, the value is 1 (True). When the value of pixel ≦ tolerance, the value of mask is 0 (False), as shown in Fig. 9c. Default tolerance is 7.
3. Find a rectangular area which includes column and row elements with 1 values (red square in Fig. 9c).
4. Extract the rectangular area from the image (RGB format).

#### Create a circular crop around the center of the fundus image

After removing the black border of the fundus image, some parts of the information are also removed, as shown in Fig. 9b, and the form of the fundus image is not circular. Even if we resize the fundus image (Fig. 9b), the fundus images will be deformed. In order to create a circular crop around the imaged center of the fundus image, as shown in Fig. 9d, this study adopts the following processing methods:

1. Find the height (H) and width (W) of the fundus image (H*W).
2. Find the longest side (L), either the height or width.
3. Resize the fundus image (Fig. 9 (b)) (L*L).
4. Produce the circular image. There is circular mask with radius (L/2) at center, where the value of the mask is one. Values outside the circular mask are zero.
5. Combine the fundus image (Fig. 9 (b)) with the circular image using cv2.bitwise_and (OpenCV).
6. Remove the black border of the fundus image again, as mentioned above.

#### Assess quality of the fundus image

**Fig. 10:**
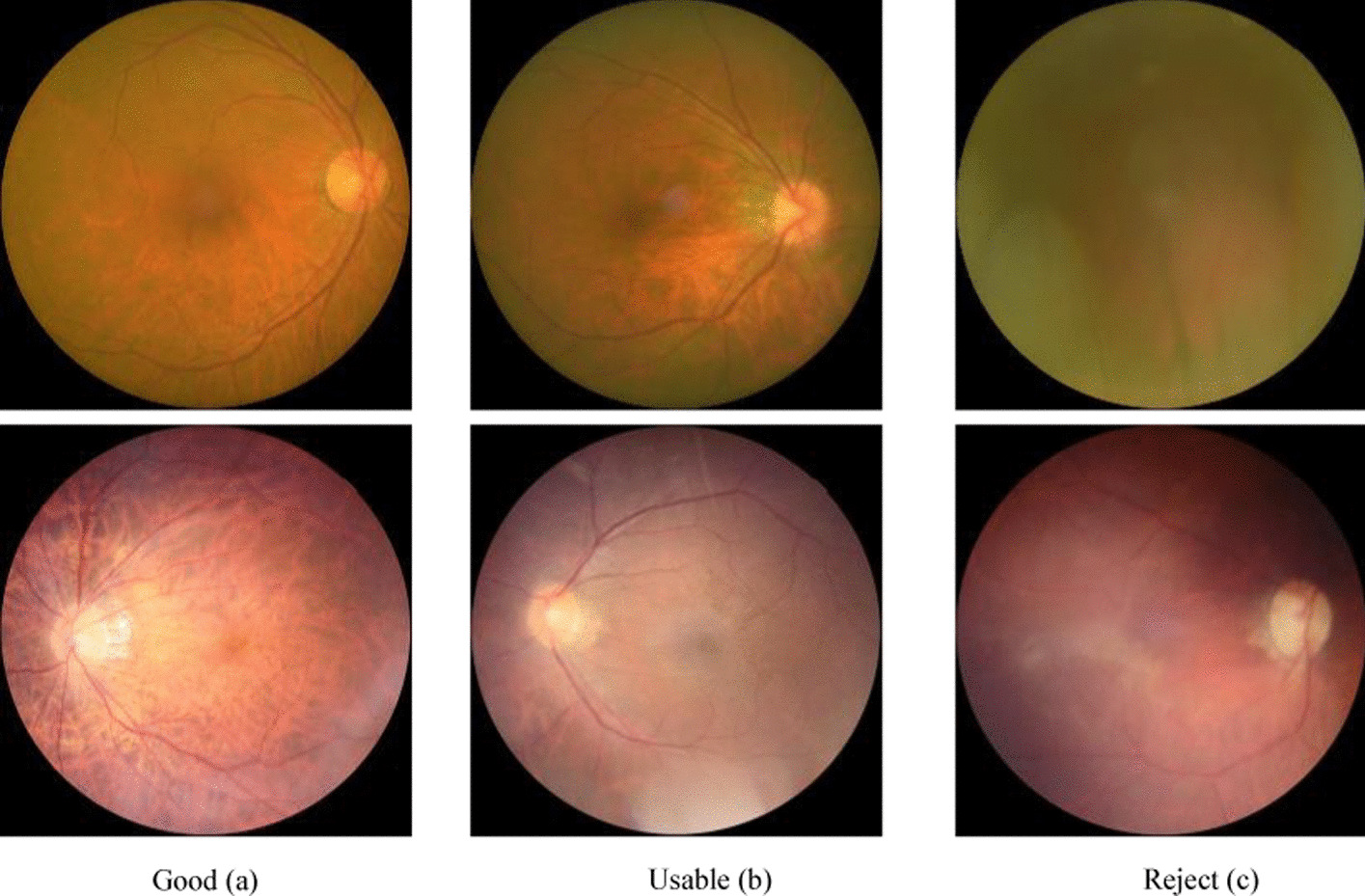
Assess the quality of fundus images using the Eye-Quality library. **a** Good, **b** Usable and **c** reject

In order to obtain the most important features from fundus images, this study adopts the Eye-Quality library [22, 23] to assess the image quality with three labels: reject, usable, and good (Fig. 10). The Eye-Quality (EyeQ) library (https://github.com/HzFu/EyeQ) is developed from the EyePACS dataset to provide fundus image quality assessment, using a multiple color-space fusion network (MCF-Net) based on ResNet121. The dataset only includes usable and good quality fundus images, to train and test the performance of DR grading.

#### Equalize the histogram of the fundus image

**Fig. 11:**
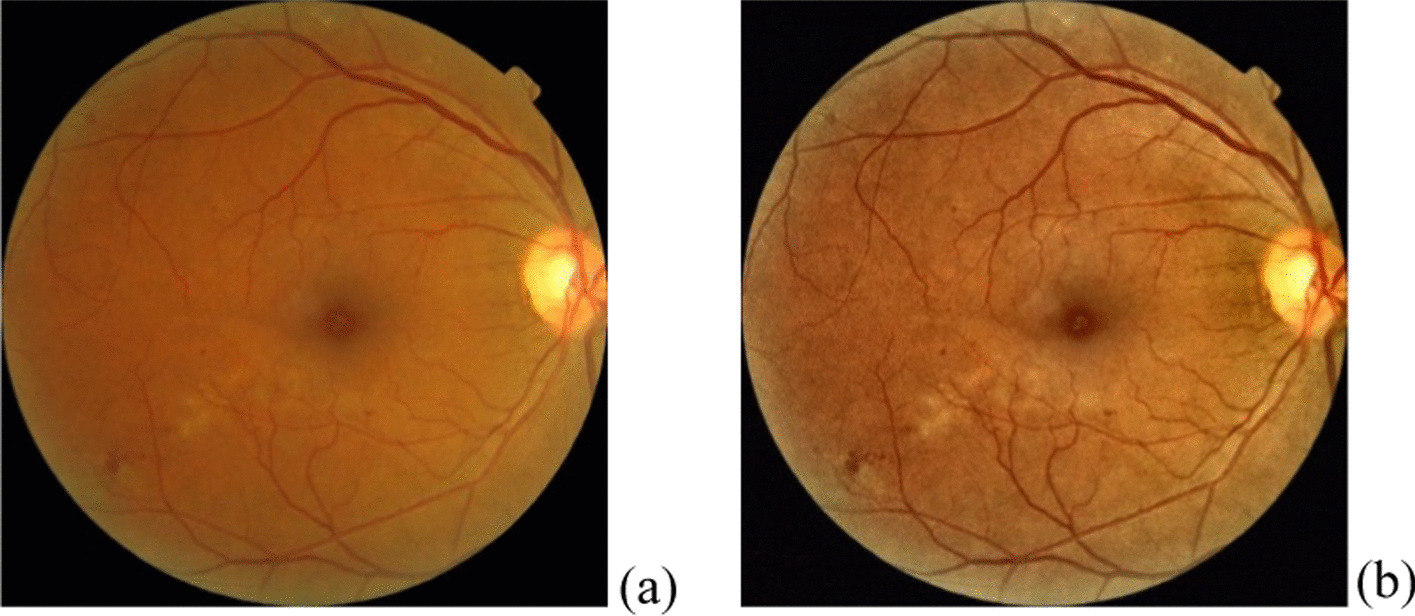
The image before equalizing (histogram **a**) and after equalizing (histogram **b**)

This study uses an equalized histogram of the fundus image, where the distribution of the image is changed to a uniform distribution, to enhance the contrast and make the features relatively clear. This study equalizes the histogram in Fig. 10, which is a good or usable image. An image in RBG format should be transferred to YCrCb or HSV format before equalizing the histogram. For YcrCb format, Y is the luma component, and Cr and Cb are the red-difference and blue-difference chroma components. HSV format is an alternative representation of the RGB color model, and has three components: hue, saturation, and value. This study transfers the image from RBG format (Fig. 11a) to HSV format first, and then equalizes the histogram of hues and values of the fundus image [24] (Fig. 11b).

### Revised structure of residual neural network (ResNets)-50

Deeper CNN with more layers suffered from the vanishing gradient problem easily. Although this problem can be solved by normalizing and intermediate initialization, the results of Deeper CNN showed a worse performance on both train and test errors, and it was not caused by the overfitting problem. In order to solve this issue, He et al. [25] adopted the pre-trained shallower model with additional layers to perform identity mapping. They then proposed a deep residual learning framework as a solution to the degradation problem. The architecture of ResNet consisted of stacked residual blocks of 3 × 3 convolutional layers. Then, the number of filters can be periodically doubled and used with a stride of 2. The first layer can utilize a 7 × 7 conv layer and didn’t feature fully connected layers at the end. There were different depth layers for ResNet: 18, 34, 50, 101 and 152. When the CNN had depth more than 50,’bottleneck’ layer can be applied for dimensionality reduction and to improve efficiency. To solve the classification problems, many different types of ResNets are used, with different numbers of layers: specifically, 18, 34, 50, 101, and 152 layers [26–31]. Sultana et al. [26] explained different CNN architectures for image classification, including MNIST [32] database of handwritten digits, 1.2 million high-resolution images of 1000 classes for ILSVRC-2010 and ILSVRC-2012 [33] etc. Salah et al. [27] adopted the CNNs to estimate the heart rate from facial videos. Zhang et al. proposed a novel algorithm using a CNN architecture, named CNN-Drug–drug interactions (DDI), to learn feature representations and predict DDIs. Xiao et al. [29] detected the position of humans in the holistic image first. Then, they take advantage of a multi-stage cascade of ResNet-50 to reason about human body joint positions and estimate the human pose. Wu et al. [30] proposed the improved ResNet-50 deep learning algorithm for identification of chicken gender. The code and dataset of this previous study was released on GitHub (https://github.com/PuristWu/Identifying-gender). Nayak et al. [31] used 2259 smartphone images of various rice (Oryza sativa) plant parts under various classes and 250 real-time validation images for classifying 12 rice diseases and nutrient deficiency symptoms. They adopted the different image segmentation techniques, optimization methods and dynamic framework rice disease and nutrient deficiency detection. Through comparison across different models for image classification with many supporting metrics, the best model for transfer learning was selected, suggesting that the ResNet50 model was the best for cloud architectures. The current deep learning framework for detecting and grading DR is ResNet-50 [8, 9]. Therefore, we can understand the diversity of ResNets-50 application to classification problems. However, the disadvantages of ResNet-50 are overfitting and fluctuations in accuracy, which affect its accuracy in detecting DR. This study proposes three strategies to improve the performance of ResNet-50, as follows:

#### Adaptive learning rate in ResNet-50

Learning rate is a particular issue in deep learning. A high learning rate causes weight updates that will be too large, and the performance of the model will oscillate over training epochs. A learning rate that is too low may never converge or may get stuck in the local solution. Thus, this study adopts the adaptive learning rate for ResNet-50, as follows:

1. Set learning rate ($lr$=0.01) and $factor(0.5)$
2. Set the low bound of $lr$, where $lr>0$
3. If the performance of ResNet-50 fails to change every two epochs, the learning rate will be adjusted according to Eq. (3):
  3 $${lr}^{^{\prime}}=lr*factor$$

#### Regularization

Regularization can be employed to minimize the overfitting of the training model [34]. There are two common methods: L1 and L2 regularization. This study applies both L1 and L2 regularization, with kernel_regularizer, which applies a penalty on the layer’s kernel [35, 36], and activity_regularizer, which applies a penalty on the layer’s output [37].

#### Obtain suitable features from conv5_block1_out and conv5_block2_out in ResNet-50

**Fig. 12:**
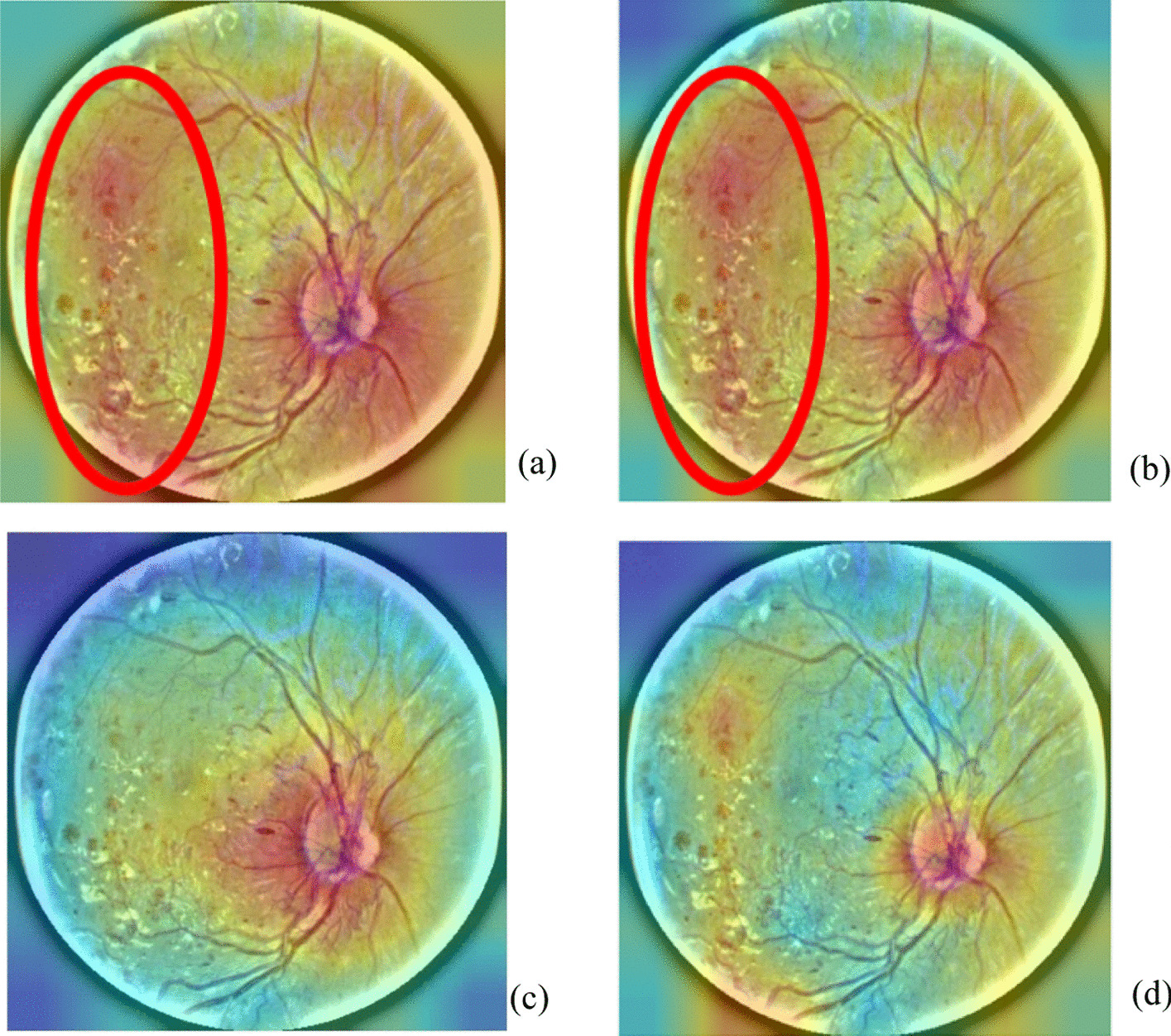
Visualization of features in the conv5_block1_out (**a**) and conv5_block2_out layers of ResNet-50 (**b**). Features from the final layer in ResNet-50 (**c**). Feature production by (a)*(b) (**d**)

A visualization tool can be applied to observe the features in different layers in ResNet-50 [38–40]. In the conv5_block1_out and conv5_block2_out layers, Fig. 12a, b shows the distinctive features, which indicate the bleeding part in red color. However, the bleeding part in red does not appear clearly in the final layer in ResNet-50 (Fig. 12c). If the features of two layers could be combined (Fig. 12d), the accuracy of DR grading should be improved. Therefore, this study performs different operations to combine the features of conv5_block1_out with those of conv5_block2_out.

### Online DR grading system

**Fig. 13:**
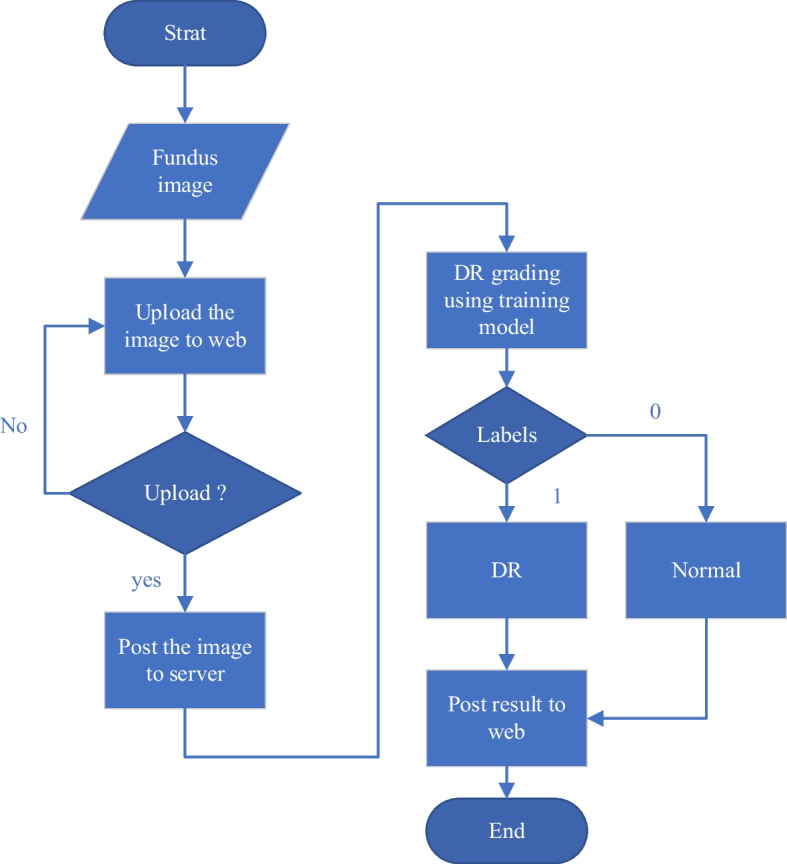
The flowchart of the online DR grading system

This study adopts Python, html and JavaScript for web development. Functions include a web application framework, sitemap management, and web interactive design. The online DR grading system can be accessed by “post,” which is a request method supported by HTTP, used by the World Wide Web as a server. Thus, users can upload their fundus image through the online DR grading system, and the training model in the server can grade the image to evaluate whether DR is present. Then, the results will be returned and shown on the website. Figure 13 shows the flowchart of the online DR grading system. The Online DR grading system was only set up in the local area network (LAN) of Ming Chi University of Technology (MCUT) to test the performance of the online DR grading system to verify that the system can detect DR conditions in real-time.

## Data Availability

The datasets used and/or analyzed during the current study available from the corresponding author on reasonable request.
